# Analysis of CPD Ultraviolet Lesion Bypass in Chicken DT40 Cells: Polymerase η and PCNA Ubiquitylation Play Identical Roles

**DOI:** 10.1371/journal.pone.0052472

**Published:** 2012-12-18

**Authors:** Ágnes Varga, Adam P. Marcus, Masayuki Himoto, Shigenori Iwai, Dávid Szüts

**Affiliations:** 1 Institute of Enzymology, Research Centre for Natural Sciences, Hungarian Academy of Sciences, Budapest, Hungary; 2 Division of Biomedical Sciences, St George's, University of London, London, United Kingdom; 3 Division of Chemistry, Graduate School of Engineering Science, Osaka University, Osaka, Japan; Institute of Molecular Genetics IMG-CNR, Italy

## Abstract

Translesion synthesis (TLS) provides a mechanism of copying damaged templates during DNA replication. This potentially mutagenic process may operate either at the replication fork or at post-replicative gaps. We used the example of T-T cyclobutane pyrimidine dimer (CPD) bypass to determine the influence of polymerase recruitment via PCNA ubiquitylation versus the REV1 protein on the efficiency and mutagenic outcome of TLS. Using mutant chicken DT40 cell lines we show that, on this numerically most important UV lesion, defects in polymerase η or in PCNA ubiquitylation similarly result in the long-term failure of lesion bypass with persistent strand gaps opposite the lesion, and the elevation of mutations amongst successful TLS events. Our data suggest that PCNA ubiquitylation promotes CPD bypass mainly by recruiting polymerase η, resulting in the majority of CPD lesions bypassed in an error-free manner. In contrast, we find that polymerase ζ is responsible for the majority of CPD-dependent mutations, but has no essential function in the completion of bypass. These findings point to a hierarchy of access of the different TLS polymerases to the lesion, suggesting a temporal order of their recruitment. The similarity of REV1 and REV3 mutant phenotypes confirms that the involvement of polymerase ζ in TLS is largely determined by its recruitment to DNA by REV1. Our data demonstrate the influence of the TLS polymerase recruitment mechanism on the success and accuracy of bypass.

## Introduction

The successful replication of damaged genomic DNA is essential for cell survival, but this process is also a main cause of mutagenesis. Translesion synthesis (TLS) is a universal lesion bypass mechanism that employs specialised reduced fidelity DNA polymerases to synthesise the new DNA strand along the damaged template [1]. Several TLS polymerases are present in higher eukaryotes, and potentially a number of them are available for the bypass of a particular type of lesion [2]. Their choice and regulated access to DNA can thus determine the outcome of TLS.

The recruitment of TLS polymerases to the site of replication stalling lesions was first suggested to occur by their binding to the monoubiquitylated form of the replication protein PCNA based on yeast experiments [3], and indeed TLS by both yeast Pol η and ζ was found to depend on this PCNA modification [4]. The Y family TLS polymerases may all directly bind PCNA-Ub, as Pols η, ι and κ as well as REV1 have been shown to contain evolutionarily conserved ubiquitin binding domains [5]. The B family polymerase Pol ζ comprises the REV3 catalytic subunit and REV7; in *S. cerevisiae* the p31 and p32 Pol δ subunits have also been shown to contribute to Pol ζ [6], [7]. Direct interactions between the Pol ζ subunits and ubiquitylated PCNA have not been reported. More recently, TLS has also been observed in the absence of PCNA ubiquitylation in yeast and higher eukaryotes [8], [9], [10]. In chicken DT40 cells the remaining TLS was shown to be coordinated by the REV1 protein [9]. REV1 is able to bind PCNA directly [11], [12], and can also bind any one of Pols η, ι, κ and ζ [13], [14], [15]. PCNA monoubiquitylation and the REV1 protein may thus form the core of two alternative mechanisms of TLS polymerase recruitment, which in some cases can recruit the same polymerase [9].

The choice of TLS polymerase recruitment mechanism has been correlated with the timing of TLS relative to the movement of the replication fork, a topic that has come under recent scrutiny [16], [17], [18]. REV1 is required for replication fork progression following UV irradiation [19], [20], suggesting that it coordinates a polymerase switch at the stalled replication fork, whereby the replicative polymerase is temporarily replaced by a TLS polymerase [21]. Also after UV irradiation, PCNA ubiquitylation is necessary for the timely appearance of high molecular weight nascent DNA [19], suggesting a role in filling post-replication gaps that arise at UV lesions. These gaps, first observed in *E. coli* after UV irradiation [22], are created by the replication fork initially bypassing the lesion and leaving a discontinuity in the nascent strand opposite the lesion. This process must take place efficiently even on the leading strand, as the progress of individual replication forks is not significantly hindered after UV damage [23]. The gaps can be filled at a later stage by a process that involves TLS polymerases [24], [25]. Gap filling can be expected to serve as a backup for failed polymerase switching. In addition, recombination-type mechanisms may also repair post-replication gaps [23], [26], [27]. It is important to experimentally address the hierarchy between the different options for lesion bypass, as the choice of bypass mechanism affects the mutagenic outcome.

Physiological DNA damage often results in a range of lesion types. It is necessary to study the created lesion types individually to investigate whether the employed bypass mechanisms are lesion-dependent or whether different mechanisms operate on the same lesion. Studying the *in vivo* bypass of a single lesion type is made possible by episomally replicating plasmids that contain defined synthetic lesions. In this study we make use of the pQ1 shuttle plasmid [9] to investigate TLS over *cis-syn* thymine dimers. This plasmid can carry synthetic lesions within a fully double stranded sequence, and replicates without the aid of viral proteins by virtue of expressing a human CDC6 protein fused to a GAL4 DNA binding domain, which binds to tandem GAL4 binding sites on the same plasmid to establish an origin of replication.

Crosslinked neighbouring pyrimidine bases in the form of *cis-syn* cyclobutane pyrimidine dimers (CPDs) are the most common UV-generated physiological DNA lesions, and the T-T form is the most frequent of these [28]. CPD lesions make the major contribution to UV-induced mutagenesis [29], [30], presumably because they are less efficiently removed by nucleotide excision repair prior to replication than the other common crosslinked pyrimidine lesions, (6–4) photoproducts. The most common type of UV induced mutations are C to T transitions at cytosine containing dipyrimidine sequences, thought to be caused by cytosine deamination in CPD lesions prior to lesion bypass (reviewed by [31]). Several other UV-induced mutation types remain unexplained, amongst these the typical A to T transversion in the likely UV-dependent common V600E BRAF mutation in malignant melanoma [32]. The *in vivo* bypass of T-T CPDs has been assayed in human NER-deficient cells, but it was found to be rarely mutagenic, with no clear mutation spectrum [33].

Here we set out to use the example of T-T CPD bypass to determine the influence of the polymerase recruitment mechanism on the efficiency and mutagenic outcome of TLS. Using mutant chicken DT40 cell lines we genetically dissected the mechanisms of T-T CPD bypass. Defects in Pol η or in PCNA ubiquitylation similarly resulted in persistent single stranded gaps at the site of the lesions and the elevation of mutations amongst successful TLS events, indicating that PCNA ubiquitylation plays an essential function in lesion bypass primarily by recruiting Pol η. In contrast, Pol ζ was observed to be responsible for the majority of CPD-dependent mutations, but had no essential function in TLS. The similarity of REV1 and REV3 mutant phenotypes in TLS efficiency and mutagenicity suggest that the involvement of Pol ζ in TLS is largely determined by its recruitment to DNA by REV1.

## Materials and Methods

### Plasmid construction

Oligonucleotides containing a *cis-syn* thymine dimer were synthesised using a previously described building block [34]. The following oligonucleotides were used: TTCPDF1 (AATTGTCCACCTC-T(*cis-syn*)T-CCTGTATTCTTAGTACCTACTGACGCTAGCTCGATCCATGCA), TTCPDR1 (TGGATCGA-T(*cis-syn*)T-TAGCGTCAGTAGGTACTAAGAATACAGGGCGAGGTGGAC), TTCPDR2 (TGGATCGAGCTAGCGTCAGTAGGTACTAAGAATACAGG-T(*cis-syn*)T-GAGGTGGAC). The inserts for the plasmids containing two lesions in staggered or opposing arrangements were annealed in pairs as follows: pQ-CPDs (TTCPDF1 and TTCPDR1), pQ-CPDo (TTCPDF1 and TTCPDR2). In lesion-free control oligonucleotides used to assemble plasmids with a lesion in only one strand, the photoproducts were replaced with the dinucleotide GC. The annealed inserts were ligated into the EcoR I and Pst I sites of pQ1 in two steps as described [35]. The products of the second ligation were purified by ethanol precipitation.

### Cell culture and transfection

DT40 cells [36] were cultured at 37°C in RPMI-1640 medium supplemented with 7% fetal bovine serum and 3% chicken serum. The mutant cell lines used in this study have been described previously: *rev1, pcna^K164R^* [37]; *xpa, xpa rev1, xpa rev3, xpa polh, xpa pcna^K164R^* [9]; *xpa polh rev3* [38]. Transient transfections were performed using an Amaxa Nucleofector device (Lonza) with reagent T according to the manufacturer's instructions. In each reaction 3 million cells were transfected with 0.2 μg purified pQ1 ligation product.

### Plasmid extraction, digest and PCR amplification

40 hours after transfection plasmids were extracted using a simplified Hirt protocol and subjected to Dpn I digestion as described [9]. 2% of the recovered plasmid mixture or defined amounts of untransfected control plasmid were used as a PCR template for the amplification of a 1369 bp region covering the sites of the lesion plus 12 Dpn I sites, using primers CAGTCGACTTTTGATTTAGAATTGTCCAC and CAGCGGCCGCTTTGCAAGCAGCAGATTACG containing Sal I and Not I sites, respectively. In case of the single strand specific nuclease digest, the same amount of recovered plasmid mixture was treated with 0.1 unit of mung bean nuclease (New England Biolabs) in 10 μl for 15 minutes at 25°C. Mock treatments were also performed. The reactions were stopped by adding 0.01% SDS, the products were purified by ethanol precipitation, and used as template for one PCR reaction. PCR reactions were performed in 20 μl volume using the Pfu Turbo polymerase (Agilent) according to the manufacturer's instructions. After a restriction digest with Sal I and Not I, the PCR product mixture was cloned into the corresponding sites in pBluescript (Stratagene), and used for transformation. Plasmid preps from individual bacterial colonies were sequenced using the M13 forward primer. As an alternative to PCR amplification, 20% of the recovered Dpn I digested pQ plasmid preparation was used to transform MegaX DH10B electrocompetent *E. coli* (Invitrogen), and plasmid preps from individual colonies were sequenced using primer GATGGTTCACGTAGTGG.

### Measurement of the rate of plasmid replication

Samples of recovered and Dpn I-digested pQ-CPDs plasmid were subjected to quantitative PCR using primer pairs pQRT1 (GAACCAACAAATGTCCAAACCG, AACAAGGAGGTAAATGGGGAGTG) and pQRTD6 (GATGGTAAGCCCTCCCGTAT, TTTGCAAGCAGCAGATTACG), the latter spanning a region that contains 6 Dpn I sites. The amount of template was quantified using standard curves obtained with pure control plasmid. The pQRTD6/pQRT1 ratio was used to calculate the percentage of Dpn I resistant, replicated plasmids.

### Cell cycle analysis

2 million cells were resuspended in 0.5 ml PBS and spread on the bottom of 6-well plates. UV irradiation was performed using a 254 nm UV lamp calibrated with a matching UV detector (both from UVP, LLC). Pre-warmed growth medium was added immediately after irradiation. Treated or untreated populations containing 1 million cells were incubated with 10 μM bromodeoxyuridine (BrdU, Sigma) for 20 minutes prior to fixation in cold 70% ethanol. The samples were rehydrated in phosphate buffered saline (PBS), treated with 2N HCl and 0.5% Triton X-100 for 30 minutes, neutralised using two PBS washes, then stained with 2 μl directly conjugated anti-BrdU-FITC antibody (eBiosciences) in 100 μl PBS/0.5% Tween-20/1% BSA for 30 minutes. After a PBS wash, cells were resuspended in PBS with 10 μg/ml propidium iodide and analysed using a Beckman-Coulter FC500 flow cytometer and the FlowJo software.

## Results

### In vivo replicative bypass of synthetic CPD lesions

**Figure 1:**
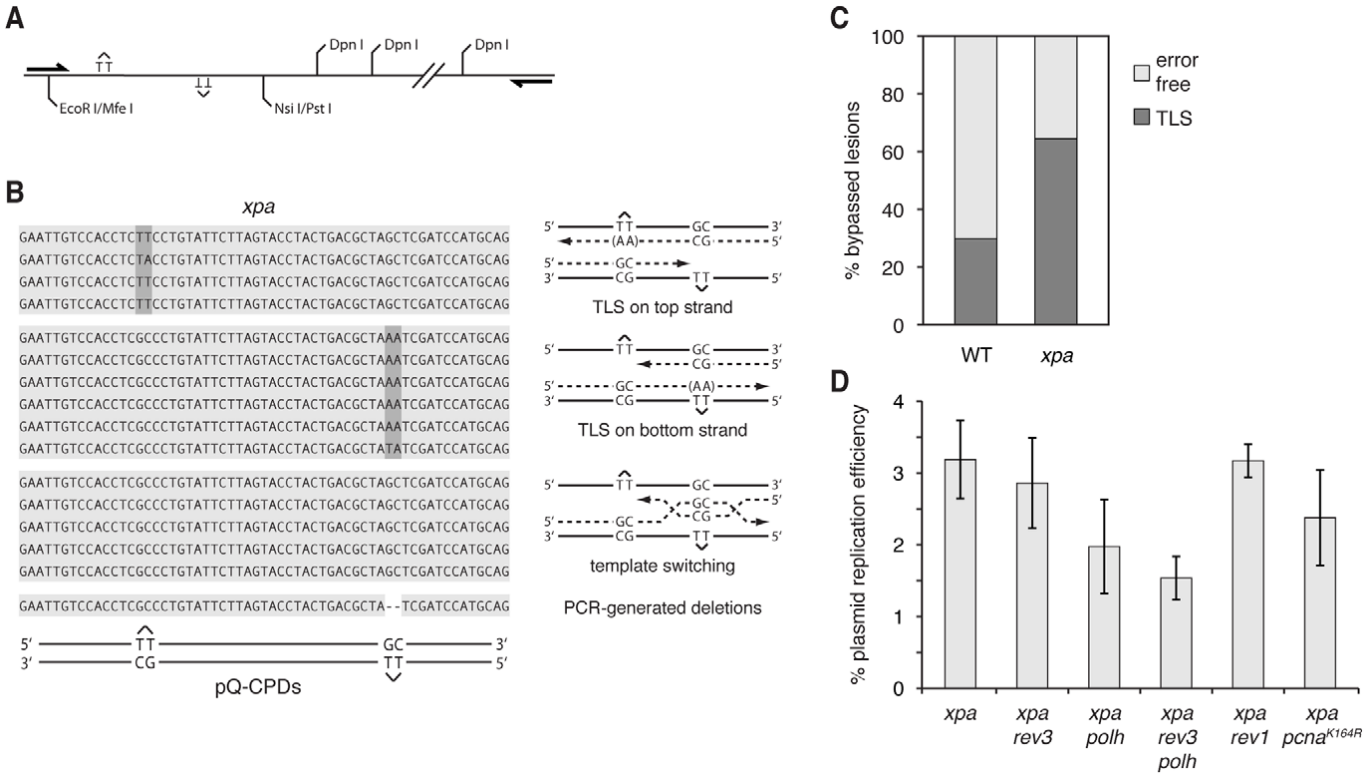
Monitoring lesion bypass in a shuttle plasmid. (A) A schematic representation of the amplified region of the pQ-CPDs shuttle plasmid. The cloning sites for the lesion-containing oligonucleotide, the lesions on each strand, and the approximate positions of the PCR primers are shown, not to scale. Some of the twelve Dpn I sites are omitted for clarity. (B) Representative sequences obtained from *xpa* cells, grouped into categories by bypass type (indicated with schematic drawings on the right). (C) The proportion of replicated plasmids recovered from wild type (WT) or *xpa* mutant cells showing evidence of translesion synthesis (dark grey) or the use of the opposite strand as a repair or bypass template (‘error free’, light grey). (D) Replication efficiency of the pQ-CPDs plasmid in the indicated cell lines, as measured by qPCR (see Materials and Methods). The mean and S.E.M. of 3–8 measurements is shown.

Synthetic T-T CPD lesions [34] were incorporated into the pQ1 shuttle plasmid, which provides a useful tool for the analysis of physiological lesion bypass [9]. The resulting fully double stranded pQ-CPDs plasmid was introduced into DT40 cells. After 40 hours the plasmids were extracted and unreplicated copies were digested using the dam methylation specific restriction endonuclease Dpn I. Dpn I resistance is widely used to detect the replication of plasmid DNA in eukaryotic cells (e.g. [39]). The lesion-containing region of the plasmid was amplified by PCR. The amplified 1369 bp region also contains 12 Dpn I sites (Fig. 1A) to ensure that all unreplicated sequences are efficiently cut prior to the PCR amplification, even if some of their Dpn I sites become resistant to the enzyme independent of replication. [40]. The sequence of individual PCR product molecules was determined after ligation into a plasmid vector and amplification in *E. coli*.

Two lesions were incorporated into the shuttle plasmid on separate strands, each opposite a GC dinucleotide, with 28 intervening base pairs. This arrangement ensures that lesion bypass must take place to generate any replicated copy. It also allows the distinction between translesion synthesis across the lesion versus bypass using the sister chromatid as an alternative template [9], [35], [41]. In *xpa* mutant DT40 cells we saw evidence of TLS over the T-T CPD, with two adenines or related base combinations appearing in the copied strand (Fig. 1B). A separate cohort of the resulting copies contained at the site of both lesions the GC dinucleotide sequence originally placed opposite the CPD lesions (Fig. 1B). The use of an *xpa* genetic background inactivates nucleotide excision repair, the repair pathway that could excise the lesions from the template prior to replication, and fill the gap using the opposite strand as template. The appearance of GC at both lesion sites therefore suggests some form of template switching, i. e. the use of the sister chromatid as template during replicative lesion bypass. In wild-type DT40 cells 30% of the successfully replicated sequences showed evidence of TLS and 70% had the sequence of the strand opposite both lesions, while in *xpa* cells these proportions change to 64% and 36%, respectively (p<0.001, Fisher's exact test; Fig. 1C and Table 1). The doubling of the TLS frequency shows that NER repairs about half of the lesion-containing plasmids prior to their replication. Subsequent transfections were therefore performed in the NER-deficient *xpa* genetic background.

**Table 1:** Type of bypass in fully replicated pQ-CPDs plasmids.

	TLS (%)	TSw (%)	Total	No. expts	p value vs. *xpa*
WT	22 (30%)	52 (70%)	74	5	<0.001
*xpa*	67 (64%)	37 (36%)	104	6	-
*xpa rev3*	45 (41%)	64 (59%)	109	6	0.001
*xpa polh*	47 (67%)	23 (33%)	70	6	0.747
*xpa polh rev3*	38 (43%)	51 (57%)	89	5	0.004
*xpa rev1*	38 (46%)	45 (54%)	83	5	0.012
*xpa pcna^K164R^*	46 (55%)	38 (45%)	84	7	0.230

The number of sequences generated by TLS or by using the opposite strand as template (template switching, TSw) is shown in different cell lines, followed by the percentage in each group. Deletion-bearing PCR products are omitted. The total number of sequences obtained (Total), and the number of independent experiments performed to generate each dataset (No. expts) is also shown. Each genotype was compared to *xpa* using Fisher's exact test; the probability values are shown in the last column.

To aid the genetic analysis of lesion bypass presented below, we first measured the rate of plasmid replication in all the cell types used by quantitative real-time PCR. One primer pair spanning a region with no Dpn I sites was used to measure the total amount of plasmid retrieved from transfected cells. A second primer pair spanning a region with six Dpn I sites measured the amount of replicated DNA, and the ratio of the replicated to total plasmid provided the rate of replication. We measured a range of mean values between 1.5–3%, with a relatively high variability between measurements (Fig. 1D). The low measured rate of replication is probably due to inefficient replication initiation promoted by the GAL4-CDC6 fusion protein expressed by the pQ plasmid. For comparison, this protein promoted 11% plasmid replication in human 293A cells, but caused no detectable plasmid replication in Drosophila S2 cells [39], [42]. The inefficient replication initiation should not interfere with the study of replicative bypass of lesions away from the replication origin. Using one-way ANOVA, the difference between mean replication rates in the mutant cell lines was not statistically significant (p = 0.22).

### The detection of incomplete CPD lesion bypass

**Figure 2:**
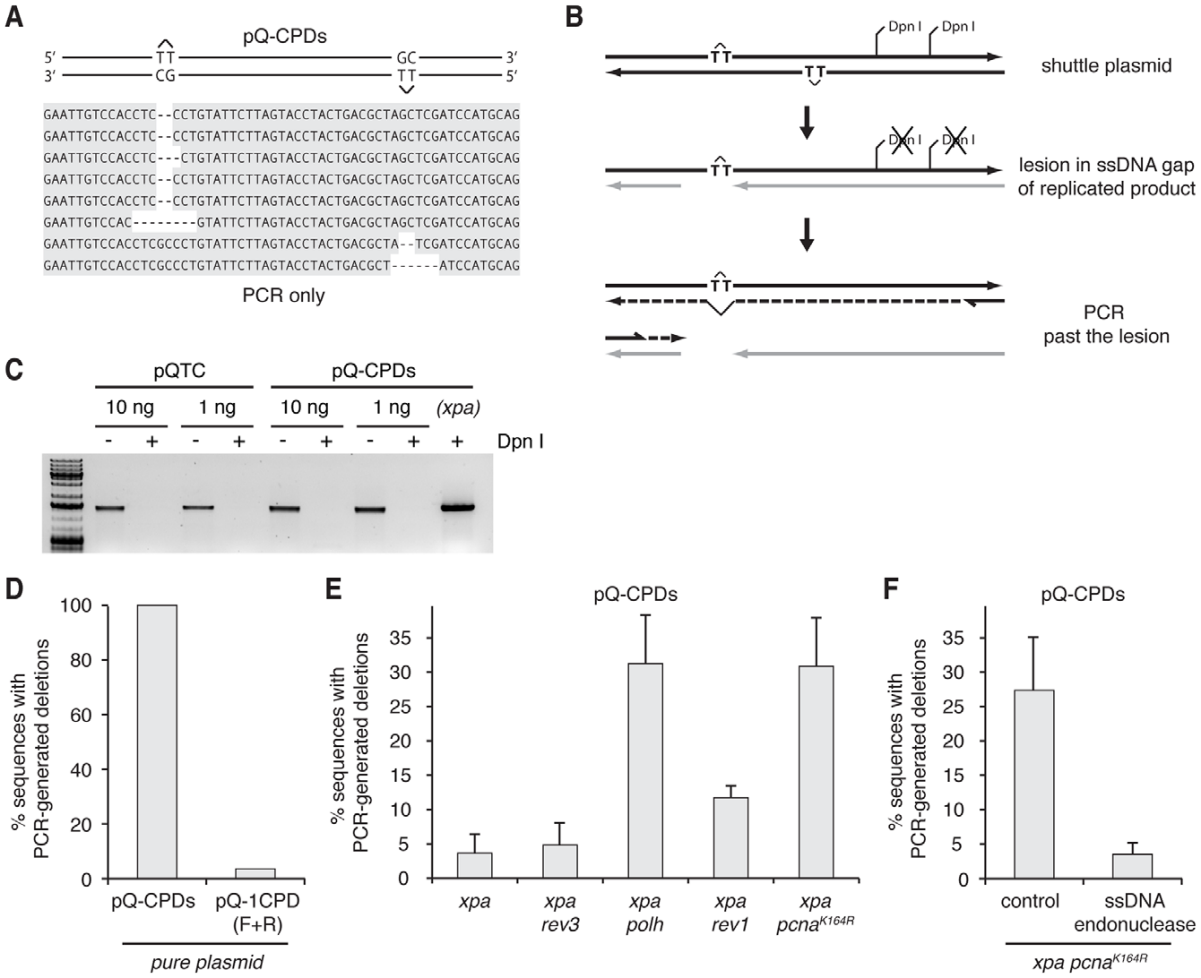
Post-replicative ssDNA regions at CPD lesions detected by PCR. (A) Representative samples of sequence deletions generated by a PCR reaction using the pure pQ-CPDs plasmid as template. (B) A schematic drawing for the generation of deletions at single-stranded gaps. Replication of a lesion-containing strand may produce a gap in the daughter strand (grey). The gapped strand is not suitable as a PCR template, so the PCR reaction initially must proceed across the lesion (dashed line). (C) Dpn I digested untransfected control pQTc and lesion-containing pQ-CPDs plasmid do not serve as PCR templates. The indicated amounts of plasmid were subjected to Dpn I digests (+) or mock digests, amplified by PCR and analysed on an agarose gel. A positive control sample extracted from *xpa* cells and subjected to the same treatment is shown in the right hand lane. (D) The percentage of deletions amongst the PCR products of pure plasmids containing CPD lesions on both strands (pQ-CPDs) or only one strand (pQ-1CPD). (E) The percentage of deletions amongst PCR products of plasmids recovered from transfected cells of the indicated genotypes. The mean percentage and S.E.M. of 3–7 experiments are shown. (F) A reduction of deletion-bearing PCR products when the plasmid recovered from *xpa pcna^K164R^* cells is pre-treated with the ssDNA specific mung bean nuclease. The mean and S.E.M. of 3 experiments are shown.

In addition to the two categories of sequences that we classified as translesion synthesis and template switching, we also detected occasional PCR products with a short, most commonly 2-base deletion at the site of one lesion (Fig. 1B). We suspected that these deletions were generated during the PCR reaction rather than during DNA replication inside the cell. To test this, we performed the identical PCR reactions on the original lesion-containing plasmid, without subjecting the plasmid to transfection or Dpn I digest. These reactions did generate a product, and sequencing individual copies of the reaction product revealed short sequence deletions in every sequence, at the site of one or the other lesion (Fig. 2A, D). This shows that the PCR polymerase (Pfu Turbo) is able to synthesise through T-T CPD lesions, but it does not insert any bases opposite the lesions, rather it makes a deletion via template slippage. To further confirm that the deletions are PCR-derived, in a separate experiment we analysed the result of lesion bypass in *xpa, xpa polh*, and *xpa pcna^K164R^* mutant cells without employing a PCR amplification step. Instead, we used the recovered and Dpn I digested plasmid to directly transform supercompetent bacteria as in our previous work [9]. Upon sequencing the plasmids extracted from transformed colonies, we never detected the deletions (Fig. S1). We therefore concluded that PCR reactions could be used to detect T-T CPD lesions within the template sequence.

CPD lesions may be found in shuttle plasmids extracted from transfected NER-deficient cells if (1) the plasmid has not replicated, (2) the plasmid replicated successfully, and retains a lesion in the template strand, (3) the replicated plasmid contains a lesion in a single stranded region. Unreplicated plasmids in scenario (1) should not form templates in our PCR reactions as they are destroyed by Dpn I. Indeed, Dpn I digests of even excess amounts of untransfected lesion-free or lesion-containing pQ plasmid preparations do not contain PCR templates under identical digest and PCR conditions (Fig. 2C). In addition, when the Dpn I digest was omitted on pQ-CPDs extracted from *xpa* cells, 20 out of 20 PCR-generated sequences had short deletions (data not shown), giving identical results to the untransfected plasmid. This shows that although plasmid replication is very inefficient, the Dpn I digest successfully prevents the PCR amplification of unreplicated plasmids. CPD lesions surviving in the template strand of replicated shuttle plasmids as in scenario (2) should give the same PCR results as pure synthetic plasmids with lesions located in only one strand. We synthesised such plasmids with only one CPD lesion, in a position equivalent to either lesion in pQ-CPDs. Averaging the results from the two lesion positions to allow for the influence of sequence context, we found PCR-generated deletions in 4% of all sequences (Fig. 2D). If the reaction proceeded with equal efficiency on the lesion-containing and the lesion-free strand, 50% of products should bear evidence of CPD lesions. PCR-mediated CPD bypass is therefore rather inefficient, and PCR reactions on replicated plasmids (with a lesion in one strand at most) can only generate a small percentage of products with deletions at the lesion site. If an increase is seen in the proportion of sequences with deletions, this should be due to the presence of single-stranded regions as mentioned in scenario (3), where the only way to form a PCR product is to proceed through the lesion (Fig. 2B). Single-stranded DNA (ssDNA) regions around the lesion could arise by a number of mechanisms, including unfinished Okazaki fragment synthesis, the re-priming of DNA replication downstream of the lesion without completing lesion bypass, or possibly if a replication fork approaching from the opposite direction copies the damaged strand downstream of the lesion. The appearance of PCR-generated deletions can be used to detect persistent post-replicative ssDNA regions generated by either mechanism. We employed this test to measure the success of TLS directly at the level of replicating lesion-containing DNA.

### Defects in polymerase η or PCNA ubiquitylation lead to incomplete CPD lesion bypass

If any polymerase is essential for CPD bypass, then the lack of these polymerases should result in persistent ssDNA at the site of the lesion. We tested mutants of two TLS polymerases in the *xpa* genetic background. We found that *xpa rev3* mutant cells, defective in the catalytic subunit of Pol ζ [6], did not show any elevation of CPD-derived deletions in replicated pQ-CPDs shuttle plasmids compared to the background level seen in *xpa* cells (Fig. 2E). However, in *xpa polh* cells mutant in Pol η, there was a marked increase in the mean proportion of sequences with deletions from 4% to 31% (p = 0.021, Student's t-test). Bearing in mind that the PCR reactions are less efficient on CP-containing initial templates, this implies that the majority of recovered replicated plasmids contain post-replicative ssDNA regions at the lesion. We can conclude that Pol η, but not Pol ζ, is an important contributor to the overall success of CPD lesion bypass.

DT40 cell lines carrying a *rev1* knockout or a K164R mutation in PCNA at the ubiquitylation target lysine residue have been employed to investigate the roles of these TLS polymerase recruitment mechanisms [19], [37]. In our experiments with the pQ-CPDs shuttle plasmid there was a small and just significant elevation in the mean proportion of deletion-containing PCR products in the case of *xpa rev1* cells to 11.6% (p = 0.049), and a much greater increase with *xpa pcna^K164R^* cells to 30% (p = 0.007, Fig. 2E). It thus appears that PCNA ubiquitylation at lysine 164 has a major role in the completion of CPD bypass similar to that of Pol η, and REV1 may also play a minor role.

Using the replicated shuttle plasmids recovered from *xpa pcna^K164R^* cells, we sought to confirm that we were indeed measuring ssDNA regions when detecting PCR-generated deletions. After the Dpn I digest we subjected the sample to a mild digestion with an ssDNA specific endonuclease, mung bean nuclease. We then performed the PCR amplification as before, and observed that the nuclease treatment reduced the proportion of deletion-containing products from 27% to 4% (p = 0.05, Fig. 2F). The CPD-dependent deletions are therefore derived from template sequences sensitive to single stranded DNA digestion, consistent with the CPD lesions being located in single stranded regions surrounded by replicated DNA.

### REV1 and REV3 play an important role in CPD lesion bypass

**Figure 3:**
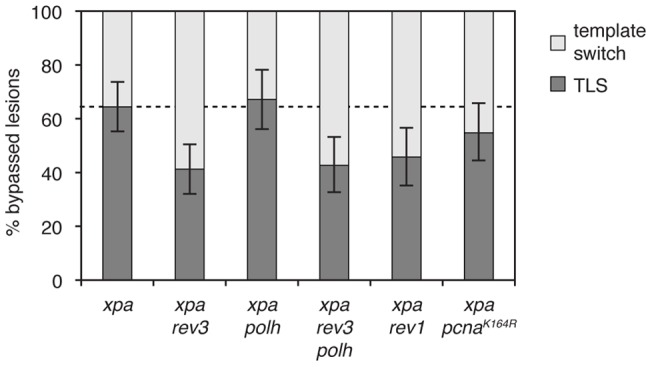
The genetic dependence of successful CPD bypass. The proportion of replicated plasmids recovered from cells of the indicated genotypes showing evidence of translesion synthesis (dark grey) or the use of the opposite strand as an alternative template (‘template switch’, light grey). Sequences with PCR-generated gaps, bearing evidence of incomplete bypass, are omitted. The level of TLS in the control *xpa* cell line is shown by a dashed line. 95% binomial confidence intervals are shown.

The main alternative to TLS in damage bypass is the employment of a template switching mechanism. We further examined the role of TLS polymerases in T-T CPD bypass through the proportion of successful bypass events that carry evidence of TLS as opposed to template switching. In *xpa rev3* mutants, PCR amplification of replicated plasmids showed a significant reduction of TLS from 64% to 41% (Fisher's exact test, p = 0.001; Fig. 3), indicating an involvement of Pol ζ in TLS over the T-T CPD. In contrast, we saw no reduction of the proportion of TLS amongst successful bypass events in *xpa polh* mutants as compared to *xpa* (p = 0.74), suggesting that the only role of Pol η lies in the avoidance of persistent post-replicative gaps on this lesion. The cell line *xpa polh rev3*, combining the disruption of polymerases η and ζ, showed 43% TLS at replicated lesions, suggesting that in the absence of Pol ζ function Pol η is still not involved in CPD bypass apart from its role at ssDNA regions.

Which mechanism is used to recruit REV3 to perform its role in TLS? We found that in *xpa rev1* mutants TLS was significantly reduced to 46% (p = 0.01; Fig. 3), a similar level as seen in *xpa rev3* mutants, suggesting that REV1 plays a role in REV3 recruitment. In *xpa pcna^K164R^* cells 55% of ssDNA-free replicated plasmids showed evidence of TLS, a non-significant reduction from the 64% seen in the control *xpa* cells. This suggests that the role of PCNA ubiquitylation is restricted to the avoidance of persistent post-replicative gaps in analogy with Pol η.

### REV1 and REV3 make the major contribution to mutagenic translesion synthesis over the T-T CPD

**Figure 4:**
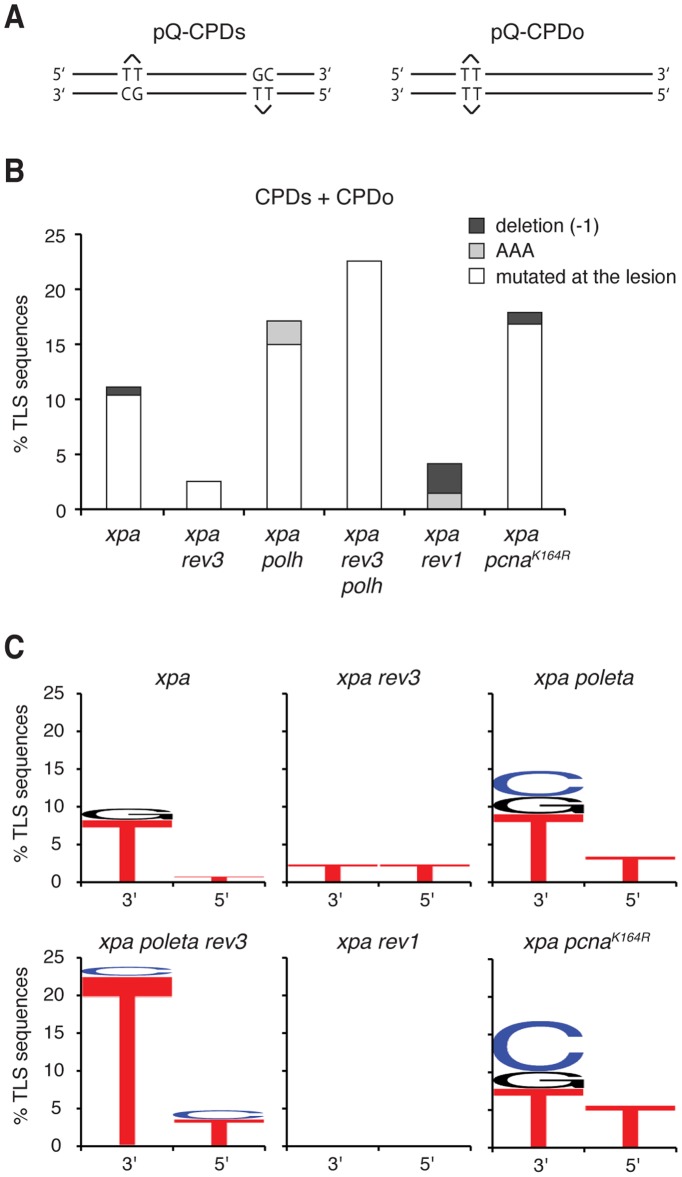
The mutagenic spectrum of CPD bypass. (A) The arrangement of lesions in the pQ-CPDs and pQ-CPDo plasmids. (B) The percentage of pooled CPDs and CPDo TLS events that are different from an AA insertion, grouped by mutation type: base change at the lesion (white), an extra A opposite the base 5′ to the lesion (AAA, light grey), one-base deletion (dark grey). (C) The frequency of different mutagenic base insertions opposite the two bases of the thymine dimer. The height of the letters indicates the frequency of occurrence of the corresponding DNA base.

CPD lesions are the major contributor to the mutagenic effect of UV [29], [30]. We asked how individual TLS polymerases, and their two separate identified recruitment mechanisms, contribute to CPD-dependent mutagenesis. The sequencing of the outcome of pQ-CPDs replication provided between 38 and 67 samples of TLS per cell line. To increase the dataset more efficiently, we transfected the same cell lines with the pQ-CPDo plasmid in which two T-T CPD lesions are placed opposite each other (Fig. 4A). In this arrangement all bypass must take place by TLS, avoiding the need to sequence template switching outcomes. We did not see any clear difference between the TLS bypass products of pQ-CPDs and pQ-CPDo, therefore the results obtained with the two constructs are presented together (Table 2). TLS of the T-T CPD mostly inserts the correct AA sequence. In all cell lines tested the most common mutations were base changes at the site of the lesion. We also occasionally observed a base change to adenine at the position immediately downstream of the lesion (an AAA insertion with no frame shift), and some single nucleotide deletions resulting from only one nucleotide inserted opposite the lesion. In *xpa* cells the overall mutation rate was 11.1%. In comparison to this, the mutation rate was reduced to 2.4% in *xpa rev3* cells and 4.5% in *xpa rev1* cells (Fig. 4B). This suggests that REV3 and REV1 make a major contribution to CPD-induced mutations. In contrast, mutagenic TLS increased to 17.0% in *xpa polh* cells and 17.9% in *xpa pcna^K164R^* cells, indicating that normally Pol η and PCNA ubiquitylation both promote TLS with a low mutation rate, and in their absence the remaining TLS is more frequently mutagenic. Finally, in *xpa rev3 polh* cells lacking both TLS polymerases investigated, an even higher level of mutations (23.2%) was observed, reflecting on the mutation spectrum of remaining polymerases capable of CPD bypass.

**Table 2:** The mutagenic spectrum of T-T CPD bypass.

TLS type	All TLS			Correct AA (%)	Mutated at lesion (%)	AAA (%)	-1 deletion (%)	Mutated total (%)	p value vs. xpa
	pQ-CPDs	pQ-CPDo	Total						
*xpa*	67	68	135	120 (88.9%)	14 (10.4%)	0 (0.0%)	1 (0.7%)	15 (11.1%)	-
*xpa rev3*	45	39	84	82 (97.6%)	2 (2.4%)	0 (0.0%)	0 (0.0%)	2 (2.4%)	0.02
*xpa polh*	47	41	88	73 (83.0%)	13 (14.8%)	2 (2.3%)	0 (0.0%)	15 (17.0%)	0.23
*xpa polh rev3*	38	44	82	63 (76.8%)	19 (23.2%)	0 (0.0%)	0 (0.0%)	19 (23.2%)	0.02
*xpa rev1*	38	28	66	63 (95.5%)	0 (0.0%)	1 (1.5%)	2 (3.0%)	3 (4.5%)	0.19
*xpa pcna^K164R^*	46	49	95	78 (82.1%)	16 (16.8%)	0 (0.0%)	1 (1.1%	17 (17.9%)	0.18

TLS sequences obtained from the pQ-CPDs and the pQ-CPDo construct are presented by mutagenic outcome. Each category is shown both as number of sequences and as percentage of total TLS. Each genotype was compared to *xpa* using Fisher's exact test with a 2×2 contingency table containing the sum of all correct *vs.* all mutated TLS events; the probability values are shown in the last column.

Detailed analysis of the mutation spectrum of TLS bypass revealed that the most common misinsertion opposite the T-T CPD is a T opposite the 3′ T of the lesion, seen in about 8% of TLS sequences in the *xpa*, *xpa polh* and *xpa pcna^K164R^* cells (Fig. 4C). The disruption of REV3 or REV1 causes this mistake to largely disappear, suggesting that REV3 and REV1 are required for this misincorporation. Interestingly, the increased bypass error rate in *xpa polh* and *xpa pcna^K164R^* mutants is mainly due to the appearance of C and some G insertions opposite the 3′ T (Fig. 4B). The analysis of mutation spectra further confirms the functional link between REV3 and REV1 on the one hand, and Pol η and PCNA ubiquitylation on the other. Taken together, our data suggest that Pol η is recruited to ubiquitinated PCNA to perform largely error-free bypass of CPD lesions, while REV3 plus REV1 perform mutagenic TLS.

### There is no detectable S phase progression following UV irradiation in cell lines defective in TLS polymerase recruitment

**Figure 5:**
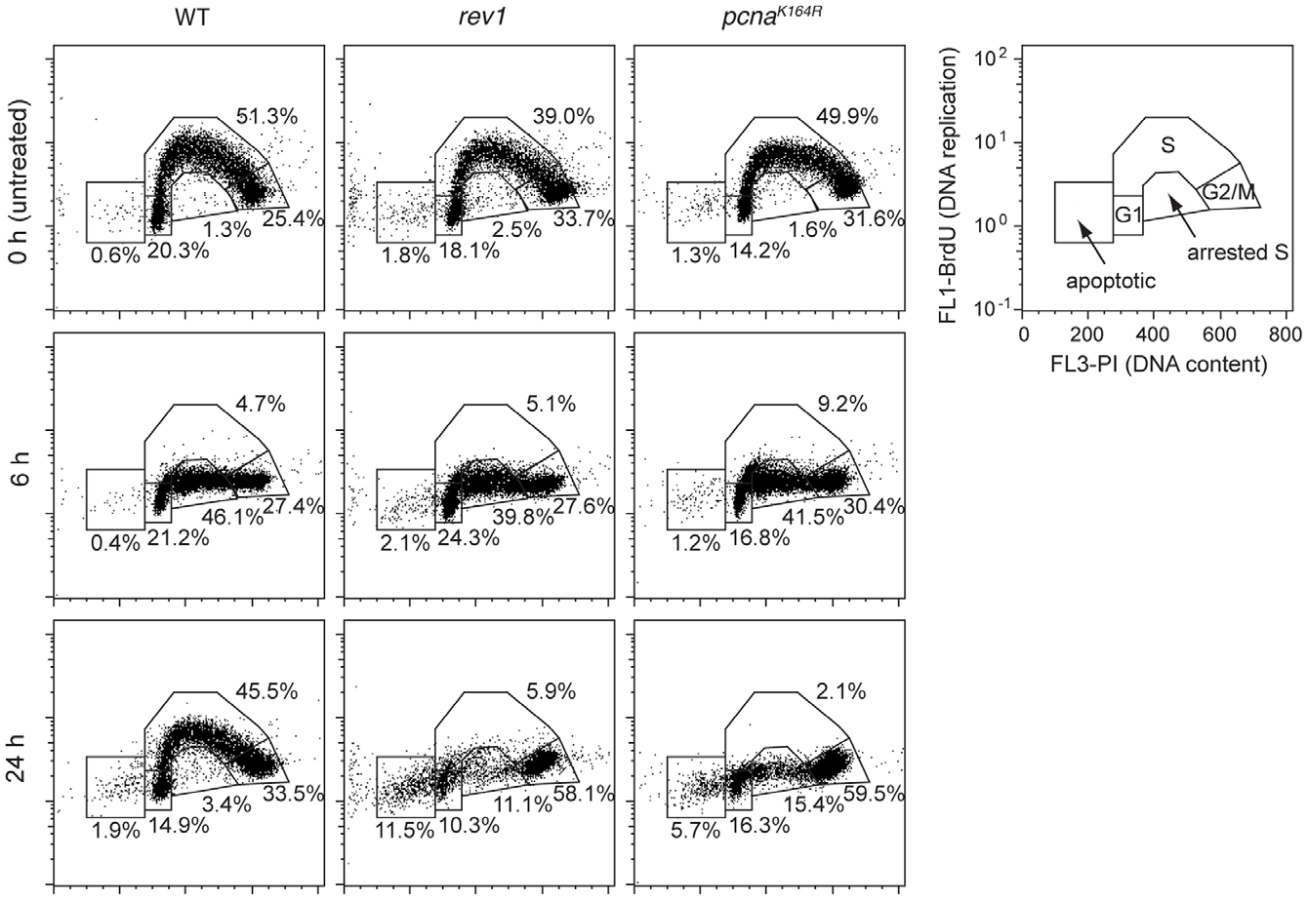
Cell cycle analysis after UV irradiation. DT40 cells of the indicated genotypes were treated with 1 J/m^2^ UV radiation, and harvested at the indicated times. The cells were treated with a pulse of BrdU before fixation. Their DNA content was analysed using propidium iodide staining (horizontal, linear axis), and their rate of DNA replication using anti-BrdU-FITC antibody staining (vertical, logarithmic axis). The separate panel on the right indicates the rationale for assigning cell populations to different cell cycle phases, including an ‘arrested S’ population with a DNA content between G1 and G2 levels but no active DNA synthesis. The percentage of cells in each category is shown on the individual panels.

We finally asked whether cell cycle progression could be used to determine whether TLS takes place concurrently with DNA replication, or in a post-replicative manner. To address this issue, we irradiated DT40 cells with 1 J/m^2^ UVC, a dose which does not significantly affect the survival of wild type cells. We did not use the *xpa* genetic background in this experiment in order to to study a more physiological response to UV radiation. We followed the fate of the cells using flow cytometry, simultaneously measuring total DNA content by propidium iodide staining and DNA replication via BrdU incorporation. 6 hours after the UV treatment there was essentially no measurable BrdU incorporation in the wild type cells. Interestingly, the cell cycle distribution did not significantly change compared to the pre-treatment, but the S phase cells stopped DNA replication (Fig. 5). The later recovery of these cells, visible at the 24 hour time point, suggests that successful lesion bypass had taken place while the cells were arrested in S phase. We looked for potential differential timing of TLS recruitment mechanisms by investigating *rev1* and *pcna^K164R^* mutants, which are mostly killed by the employed UV dose [19]. In these mutants we also observed stopped DNA synthesis in S phase cells after treatment, but we saw no resumption of DNA synthesis after 24 hours. We did however see a reduction in the arrested S phase population at 24 hours, and an increase in the relative G2/M and apoptotic populations in both mutants (Fig. 5), which could be explained by the selective apoptosis of cell arrested in S phase or the slow ‘creep’ of S phase arrested cells into G2 with BrdU incorporation levels so low as to be undetected. In any case, we did not observe any obvious difference in the reaction of *rev1* or *pcna^K164R^* mutant cells to UV treatment. This suggests that at these levels of UV damage PCNA ubiquitylation dependent post-replication repair is required during S phase and cannot take place exclusively in G2.

## Discussion

### Pol η and PCNA ubiquitylation are essential for post-replication repair

We established a method to detect the presence of T-T CPD lesions in shuttle plasmids recovered from transfected cells, and found evidence that in *xpa polh* and *xpa pcna^K164R^* mutant cells CPD lesions are located in single stranded regions of replicated plasmids. These single-stranded regions may be thought of as ssDNA gaps in an otherwise replicated plasmid. They are likely to represent post-replicative daughter strand gaps of the type that have been observed at the whole genome level by alkaline sucrose gradient sedimentation following UV irradiation in a range of organisms from bacteria to mammalian cells [22], [43], which could arise by re-priming on the leading strand or unfinished Okazaki fragment synthesis on the lagging strand. Alternatively, they may also be the consequence of two replication forks approaching from separate directions both arresting at the lesion, a situation that may more easily arise on a plasmid due to the proximity of the replication origin in either direction. In either case, the persistent ssDNA regions in the replicated plasmids point to the failure of post-replication repair in these mutants.

No significant differences were found in the overall rates of plasmid replication in different cell lines. The three detected replication outcomes (TLS, the use of an alternative template, or failed post-replication repair with ssDNA gaps at CPD lesions) therefore represent all the possible fates of the transfected plasmids. Considering that on template DNA with a lesion in one strand we saw only 4% CPD-derived product, it is clear that lesion-derived sequences are greatly underrepresented amongst the final PCR products. In accordance with this, in quantitative PCR reactions we estimated that Pfu Turbo is at least ten times less efficient when copying a CPD-containing template (data not shown). Plasmids from *xpa* or *xpa rev3* cells also give 4% CPD-derived product, which would be consistent with one round of plasmid replication resulting in a lesion in one strand, and no post-replicative ssDNA gaps. It is not possible to perform precise calculations on the overall proportion of failed lesion bypass due to the number of discrete sequences the data are derived from. Nevertheless, according to our estimates the 30% deletion-bearing PCR products indicate that perhaps 80%, and definitely well over half of plasmids recovered from *xpa polh* or *xpa pcna^K164R^* cells contained post-replicative ssDNA regions. This finding demonstrates the essential role of both Pol η and PCNA ubiquitylation in the bypass of CPD lesion.

The increased frequency of post-replicative ssDNA gaps in the mutant cell lines might be the consequence of more gaps generated, or a failure of gap filling. More than a cell cycle passed before plasmids were extracted from the transfected cells, thus it is unlikely that gaps would remain unfilled if the required mechanism were functional. Therefore, the most likely defect of *xpa polh* or *xpa pcna^K164R^* is in filling the observed post-replicative ssDNA gaps. This agrees with the post-replication repair defect of UV-irradiated Pol η defective cells from patients with the variant form of Xeroderma Pigmentosum [44], and identifies the T-T CPD as a UV lesion that requires Pol η for its successful post-replicative bypass.

In addition to TLS, the sequence data also indicate frequent CPD lesion bypass that uses the sister chromatid as template. This could either occur by the template switching mechanism that in budding yeast is reliant on PCNA polyubiquitylation and RAD5 [3], [45], or by RAD51-dependent homologous recombination. There is a general failure of post-replication repair with persistent gaps at the majority of lesions in both *xpa polh* and *xpa pcna^K164R^*, but importantly there is no major change in the TLS to template switching ratio of the successful bypass events compared to the *xpa* control cell line. This suggests several interesting conclusions. First, TLS on CPD lesions is possible in the absence of Pol η and PCNA ubiquitylation. Second, although mono- and polyubiquitylation are both impossible on the K164R point mutant of PCNA [3], [46], some damage bypass can still take place using an alternative template. This could potentially utilise a recombination pathway. Third, the unsuccessful bypass events in *xpa polh* and *xpa pcna^K164R^* also must include both failed TLS and failed template switching, and Pol η and PCNA ubiquitylation are required for both of these types of bypass. The data are consistent with PCNA monoubiquitylation promoting TLS, and PCNA polyubiquitylation promoting template switching. The requirement for Pol η in mechanisms using an alternative template is more surprising. If such template switching takes place post-replicatively, it must proceed through a strand invasion mechanism [47]. Here Pol η could perform a D-loop extender function [48] as part of a gene conversion type mechanism, as observed genetically in the chicken immunoglobulin locus [49].

### The mutagenicity of CPD bypass

In the control *xpa* cell line approximately 10% of all TLS events resulted in incorrect base insertions, a relatively high error rate when compared with similar shuttle plasmid based measurements in *E. coli* (7%), *S. cerevisiae* (0%) or human cell lines (2–4%) [33], [50], [51], [52]. The replicating plasmid used in our studies provides a more physiological system for studying events happening at the replication fork than the T antigen driven replication [33] or the non-replicating gapped plasmid [52], as bypass happens at a functional replication fork that contains only endogenous cellular proteins. Therefore the observed 10% value may be closer to the mutagenicity of genomic TLS on the T-T CPD in higher eukaryotes. The mutations we found are mostly T misincorporation events opposite the 3′ T of the lesion. This is also the most common mutagenic bypass event in *E. coli* [50]. No clear spectrum emerged in most previous eukaryotic studies due to the rarity of mutations, but the same base substitution is also the most common one in T-T CPD bypass in Pol η defective XPV cells [53]. The overall UV ‘signature’ mutation spectrum contains few A to T transversions. However, all these are found opposite dipyrimidine sequences [54], and our findings suggest that they are generated at CPD lesions.

The nearly error free TLS in *xpa rev3* mutants, and the increased error rate in the *xpa polh* mutant suggest that Pol η dependent TLS is largely error free, in accordance with earlier *in vitro* and *in vivo* observations [33], [52], [55]. The data also imply that in control cells Pol ζ is responsible for most T-T CPD-induced mutations. It is worth noting that as most UV mutagenesis is due to cytosine deamination in CPDs, we are not suggesting that Pol ζ has a role in the majority of UV induced mutations. It is interesting how Pol ζ function can lead to mutations at the first (3′) base of the lesions. Yeast Pol ζ was variously reported to have low or no CPD bypass activity *in vitro* [6], [56], and a recent study found that wild-type yeast Pol ζ is almost entirely incapable of insertion opposite the 3′ T under physiological nucleotide concentrations [57]. Pol ζ is therefore likely to fulfill an ‘extender’ function, continuing DNA synthesis from mismatched primer-template termini created by a nucleotide insertion opposite the lesion by a different polymerase [58]. Which polymerase may have the inserter function at the 3′ T? While on the T-T (6–4) photoproduct it normally seems to be Pol η [38], this is unlikely to be the case on the CPD as the A to T mutations did not change in the Pol η mutants. Chickens do not have Pol ι, therefore the best candidate for pairing with Pol ζ in the CPD bypass is the Y family polymerase Pol κ. Indeed, in the absence of both Pols η and ζ the remaining mutation spectrum is still similar, albeit at an elevated rate, suggesting that the remaining polymerases are responsible for the typical A to T mutations. Apart from Pol κ, further polymerases that have been implicated in CPD bypass are Pol μ [59], Pol β [60] and the replicative Pol δ [27].

### Polymerase recruitment and TLS timing

We genetically examined the role of PCNA ubiquitylation versus REV1 in TLS polymerase recruitment. The identical *polh* and *pcna^K164R^* phenotypes in post-replication repair defects, mutation rate and mutation spectrum imply that Pol η is recruited to CPD lesions exclusively via PCNA ubiquitylation, binding to monoubiquitylated PCNA through its ubiquitin- and PCNA-binding domains [61], [62]. The REV1 and REV3 phenotypes showed strong similarity in the reduction of TLS amongst replicated sequences, and the near elimination of mutagenic TLS. REV1 binds both the REV7 and REV3 subunits of Pol ζ [63], [64]. Therefore our results suggest a REV1-mediated recruitment of Pol ζ in agreement with the original observations in budding yeast that UV induced mutagenesis depends on both REV1 and REV3 [65], and demonstrate this mutagenic function specifically for CPD lesions.

What determines the choice of TLS polymerase and recruitment mechanism, and therefore ultimately the mutagenic or non-mutagenic outcome? The timing of TLS with respect to the movement of the replication fork may play an important role. Based on measurements of fork movement rates and nascent strand fragment size following UV irradiation, it has been suggested that REV1 directs TLS at the fork in a polymerase switching mechanism, while PCNA ubiquitylation promotes post-replicative gap filling [19]. Our results fully support this proposed role of PCNA ubiquitylation. We saw a moderate post-replication repair defect in the *xpa rev1* mutant but not in *xpa rev3*, thus REV1 may promote ‘on-the-fly’ TLS by Pol ζ and also contribute to gap filling by recruiting a different polymerase. Reports of post-replication repair defects in UV irradiated REV1 and REV3 mutant mouse embryonic fibroblasts [20], [66] and RPA foci persisting longer in G2 phase in UV irradiated REV3L knockdown U2OS cells [67] go against the model of REV1 and Pol ζ acting at the replication fork. These findings could be explained by a requirement for Pol ζ at post-replicative gaps at non-CPD UV lesions, likely (6–4) photoproducts where it plays an essential role [9].

Post-replication repair may occur either in S or in G2 phase of the cell cycle. Although several recent studies demonstrated TLS separated from DNA replication and taking place in the G2 phase of the cell cycle [24], [67], [68], we did not find a G2/M phase enrichment of UV-irradiated cells. Instead, the UV damage halted DNA replication, probably due to a checkpoint response. In addition, our data are consistent with the death of TLS defective cells specifically in S phase, suggesting that both REV1 dependent and PCNA ubiquitylation dependent damage bypass can act in S phase to aid survival and release from cell cycle arrest.

## Supporting Information

Figure S1 **Sequences derived from directly transformed replicated plasmids.** Replicated pQ-CPDs plasmid was used for the transformation of *E. coli* after extraction from DT40 cells and Dpn I digest. Plasmid extracted from antibiotic resistant bacterial colonies was sequenced to determine the outcome of lesion bypass. (A) Sequences obtained using the indicated cell types are classified according to the type of bypass event: TLS (translesion synthesis), TSw (template switching) or potential sequence deletions at the lesion. (B, C, D) Sample sequences obtained from this experiment using xpa, xpa polh, xpa pcnaK164R cells. The arrangement of the lesions in the pQ-CPDs construct is shown beloweach set of sequences. The difference between this direct transformation procedure versus the prior PCR amplification in terms of sequences with deletions at the lesion is very significant (p<0.01) according to Fisher's exact test in case of the xpa polh and xpa pcnaK164R cell lines.
(PDF)
