# Supplementary material for: Analysis of CPD Ultraviolet Lesion Bypass in Chicken DT40 Cells: Polymerase η and PCNA Ubiquitylation Play Identical Roles
Source: PLoS One. 2012 Dec 18;7(12):e52472. doi: 10.1371/journal.pone.0052472 (PMC3525536; doi:10.1371/journal.pone.0052472)
Supplement: Figure S1 — Sequences derived from directly transformed replicated plasmids. Replicated pQ-CPDs plasmid was used for the transformation of E. coli after extraction from DT40 cells and Dpn I digest. Plasmid extracted from antibiotic resistant bacterial colonies was sequenced to determine the outcome of lesion bypass. (A) Sequences obtained using the indicated cell types are classified according to the type of bypass event: TLS (translesion synthesis), TSw (template switching) or potential sequence deletions at the lesion. (B, C, D) Sample sequences obtained from this experiment using xpa, xpa polh, xpa pcnaK164R cells. The arrangement of the lesions in the pQ-CPDs construct is shown beloweach set of sequences. The difference between this direct transformation procedure versus the prior PCR amplification in terms of sequences with deletions at the lesion is very significant (p<0.01) according to Fisher's exact test in case of the xpa polh and xpa pcnaK164R cell lines. (PDF) [file pone.0052472.s001.pdf]

A

cell type	TLS	TSw	Deletion	Total
xpa	12	6	0	18
xpa polh	11	9	0	20
xpa pcna^{K164R}	11	8	0	19

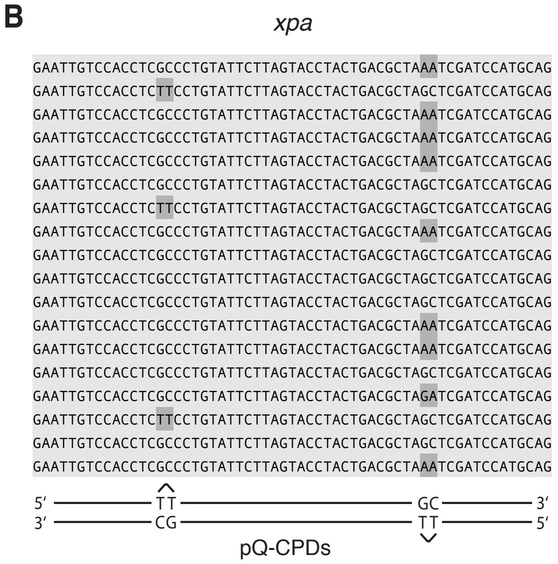

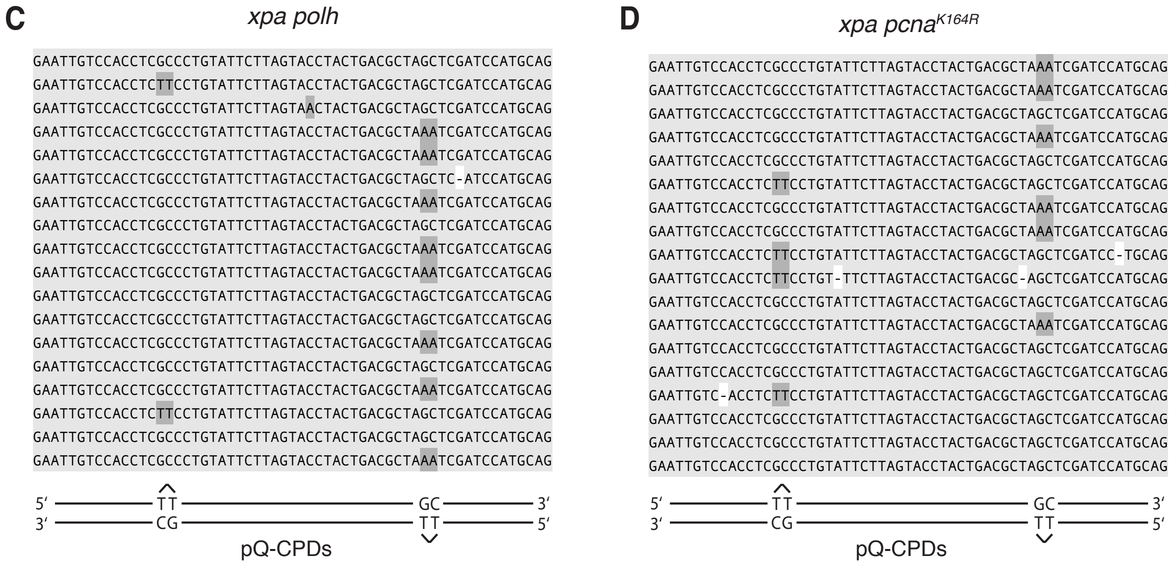

Supplementary Figure S1. Sequences derived from directly transformed replicated plasmids.

Replicated pQ-CPDs plasmid was used for the transformation of *E. coli* after extraction from DT40 cells and Dpn I digest. Plasmid extracted from antibiotic resistant bacterial colonies was sequenced to determine the outcome of lesion bypass. (A) Sequences obtained using the indicated cell types are classified according to the type of bypass event: TLS (translesion synthesis), TSw (template switching) or potential sequence deletions at the lesion. (B, C, D) Sample sequences obtained from this experiment using xpa, xpa polh, xpa pcnaK164R cells. The arrangement of the lesions in the pQ-CPDs construct is shown below each set of sequences. The difference between this direct transformation procedure versus the prior PCR amplification in terms of sequences with deletions at the lesion is very significant ($p<0.01$) according to Fisher’s exact test in case of the xpa polh and xpa pcnaK164R cell lines.
